# LHX2 facilitates the progression of nasopharyngeal carcinoma via activation of the FGF1/FGFR axis

**DOI:** 10.1038/s41416-022-01902-7

**Published:** 2022-07-21

**Authors:** Tao Xie, Kunpeng Du, Wei Liu, Chunshan Liu, Baiyao Wang, Yunhong Tian, Rong Li, Xiaoting Huang, Jie Lin, Haifeng Jian, Jian Zhang, Yawei Yuan

**Affiliations:** Department of Radiation Oncology, Affiliated Cancer Hospital and Institute of Guangzhou Medical University, Guangzhou Province, People’s Republic of China

**Keywords:** Cancer microenvironment, Cancer microenvironment

## Abstract

**Background:**

Distant metastasis and recurrence remain the main obstacle to nasopharyngeal carcinoma (NPC) treatment. However, the molecular mechanisms underlying NPC growth and metastasis are poorly understood.

**Methods:**

LHX2 expression was examined in NPC cell lines and NPC tissues using quantitative reverse transcription-polymerase chain reaction, western blotting and Immunohistochemistry assay. NPC cells overexpressing or silencing LHX2 were used to perform CCK-8 assay, colony-formation assay, EdU assay, wound-healing and invasion assays in vitro. Xenograft tumour models and lung metastasis models were involved for the in vivo assays. The Gene Set Enrichment Analysis (GSEA), ELISA assay, western blot, chromatin immunoprecipitation (ChIP) assay and Luciferase reporter assay were applied for the downstream target mechanism investigation.

**Results:**

LIM-homeodomain transcription factor 2 (LHX2) was upregulated in NPC tissues and cell lines. Elevated LHX2 was closely associated with poor survival in NPC patients. Ectopic LHX2 overexpression dramatically promoted the growth, migration and invasion of NPC cells both in vitro and in vivo. Mechanistically, LHX2 transcriptionally increased the fibroblast growth factor 1 (FGF1) expression, which in turn activated the phosphorylation of STAT3 (signal transducer and activator of transcription 3), ERK1/2 (extracellular regulated protein kinases 1/2) and AKT signalling pathways in an autocrine and paracrine manner, thereby promoting the growth and metastasis of NPC. Inhibition of FGF1 with siRNA or FGFR inhibitor blocked LHX2-induced nasopharyngeal carcinoma cell growth, migration and invasion.

**Conclusions:**

Our study identifies the LHX2-FGF1-FGFR axis plays a key role in NPC progression and provides a potential target for NPC therapy.

## Introduction

Nasopharyngeal carcinoma (NPC), a malignant tumour arising from the nasopharynx epithelium, has the highest incidence rate in Southeast Asia, especially in Southern China [1]. Most patients diagnosed with NPC are in advanced stages, and ~30% of them eventually develop distant metastasis [2]. Although, advances in intensity-modulated radiotherapy and a broader application of chemotherapy have improved the local and regional control of NPC but recurrence and distance metastasis eventually lead to treatment failure [3, 4]. Therefore, a better understanding of the molecular mechanisms that underly the recurrence and metastasis of NPC is essential in developing efficient therapeutic strategies.

Aberrant oncogenes expression universally exists in NPC and is a key feature in cancer initiation and progression [5]. However, the lack of reliable biomarkers for early detection and survival prediction of NPC limits NPC therapy. The development of high-throughput technologies and bioinformatics have identified candidate biomarkers that play important roles in NPC development, such as Serine Peptidase Inhibitor Kazal Type 6 (SPINK6), HOP Homeobox (HOPX) and Shisa Family Member 3 (SHISA3) [6–8], and transcription factors (TFs) that may offer therapeutic targets. The LIM-homeodomain transcription factor 2 (LHX2) was first identified as a critical transcription factor for B and T-lymphoid cell line differentiation. LHX2 has important roles in the development of the eye, bone, forebrain and olfactory sensory neurons [9–12]. Recent studies revealed LHX2 promotes tumour development and is highly expressed in a variety of human cancer types, including pilocytic astrocytoma, chronic myelogenous, leukaemia and pancreatic cancer [13–15]. However, the role of LHX2 in NPC is not fully understood.

Secreted proteins, such as transforming growth factor-β (TGFβ), Wnt family member 5A (Wnt5A) and epidermal growth factor (EGF), are dysregulated in the microenvironment of NPC and promote the growth, epithelial–mesenchymal transition (EMT) and metastasis of NPC in an autocrine or paracrine manner [16–18]. Fibroblast growth factor (FGF) signalling is fundamental for a variety of biological processes, including cell growth, tissue repair and tumour metastasis [19]. FGF1 is a member of the FGF family and stimulates downstream signalling cascades via binding and phosphorylating the FGF receptor (FGFR). Accumulating evidence indicates that FGF1 promotes tumour development by facilitating cell proliferation, migration, invasion and angiogenesis [20–22]. Aberrant expression of FGF1 promotes the development of various human cancers, such as breast cancer, bladder cancer and hepatocellular carcinoma [23–27]. However, the underlying role of FGF1 in NPC progression remains elusive.

In this study, we find LHX2 expression is upregulated in NPC and associated with poor survival. LHX2 promotes cellular growth, migration and invasion via promoting the FGF1 transcription, which further activates the STAT3, ERK and AKT signal pathways in an autocrine and paracrine mechanism.

## Materials and methods

### Cell culture and clinical specimens

Six human NPC cell lines (SUNE1, CNE1, CNE2, 5–8F, 6-10B and HONE1) were cultured in RPMI-1640 (Invitrogen, USA) supplemented with 10% foetal bovine serum (FBS; Gibco). The normal nasopharyngeal epithelial cell line, NP69, was cultured in keratinocyte/serum-free medium (Invitrogen) supplemented with bovine pituitary extract (BD Bioscience, San Diego, CA, USA). In all, 16  fresh frozen NPC biopsy samples and 6 normal nasopharyngeal epithelial tissues and 98 paraffin-embedded (FFPE) NPC tissues were collected from the Affiliated Cancer Hospital and Institute of Guangzhou Medical University. None of the patients had received anti-tumour treatment before biopsy collection. The study was approved by the Institutional Ethical Review Board of the Affiliated Cancer Hospital and Institute of Guangzhou Medical University, and informed consent was obtained from all patients.

### RNA extraction, reverse transcription and real-time RT-PCR

Total RNA was extracted using TRIzol reagent (Invitrogen) and reverse-transcribed into cDNA using a Reverse Transcription Kit (Promega, Madison, WI). Quantitative PCR reactions were performed using SYBR Green Real-Time PCR Master Mix Kit (Invitrogen). The experiment was performed in triplicate and the relative expression was calculated with the 2^−ΔΔCT^ method. GAPDH was applied as endogenous controls. Primer sequences are shown in Supplementary Table 2.

### Western blot analysis

Protein was extracted with lysis buffer (Beyotime, Shanghai, China) and then quantified by BCA Protein Quantitation Kit (Beyotime, Shanghai, China). Equal amounts of protein (30 μg) were separated by SDS-polyacrylamide gel electrophoresis (SDS-PAGE) and transferred to polyvinylidene fluoride (PVDF) membranes (Millipore, Billerica, MD) Later on. The membrane was blocked with 5% bovine serum albumin (BSA) and incubated with primary antibodies overnight at 4 °C. After incubation with secondary antibodies for 1 h at room temperature, the proteins were detected by enhanced chemiluminescence reagents. Primary antibodies against β-catenin (51067-2-AP), GAPDH (60004-1-Ig), and E-cadherin (20874-1-AP) were purchased from Proteintech Group. Antibodies against LHX2 (ab243030), Vimentin (ab8978), FGF1 (ab179455), ZEB1 (ab203829), FGFR1 (ab76464), FGFR2 (ab109372), FRS (ab183492) and TWIST1 (ab50581) were purchased from Abcam. Antibodies against STAT3 (#9139), Phospho-STAT3 (#9145), ERK (#4695), Phospho-ERK (#4370), AKT (#2920), Phospho-AKT (#4060), Phospho-GSK3 (#5558), Phospho-FGFR (#3471) and Phospho-FRS2 (#3861) were purchased from Cell Signalling Technology.

### Immunohistochemistry assay (IHC)

Immunohistochemistry assay (IHC) was performed on paraffin-embedded sections of clinical NPC tissues and xenograft mice tissues. In brief, the tissues were deparaffinized with xylene and rehydrated in a graded ethanol series; the endogenous peroxidase activity was blocked with 3% (v/v) hydrogen peroxide; and the slides were next operated for antigen retrieval under the citrate-mediated high-temperature. The samples were washed once with PBS, and incubated with the primary antibodies at 4 °C overnight. The sections were washed with PBS, incubated with a peroxidase-conjugated secondary antibody, and stained with diaminobenzidine (DAB). The images were captured and scored by two experienced pathologists. The staining intensity was scored as follows: 0, no staining; 1, weak, light yellow; 2, moderate, yellow brown; and 3, strong, brown. Percentage of positive cells was evaluated as: 0, negative; 1, 1–25%; 2, 26–50%; 3, 51–75% and 4, 76–100%. The final staining score was calculated by multiplying the intensity and percentage scores. The antibodies for immunostaining assay were as follows: LHX2 (1:250, Abcam, ab243030, UK), FGF1(1:250, Abcam, ab179455, UK), Ki67 (1:300, Abcam, ab16667, UK), E-cadherin (1:300, Proteintech, 20874-1-AP, USA) and Vimentin (1:200, Abcam, ab8978, UK), Phospho-STAT3 (1:200, CST, #9145), Phospho-ERK (1:200, CST, #4370), Phospho-AKT (1:200, CST, #4060) according to the manufacturer’s instructions.

### Lentiviral construction and transduction

The full length of LHX2 and FGF1 were synthesised and cloned into the lentiviral plasmid, pSin-EF2-puromycin (Addgene, Cambridge, MA, USA), and pSin-EF2-LHX2/FGF1-puromycin or the negative control pSin-EF2-puromycin vector was then co-transfected into 293T cells with the VSVG and PSPAX packaging plasmid (Addgene, Cambridge, MA, USA) using Lipofectamine 3000 Transfection Reagent (Invitrogen). The supernatant was obtained and used to infect HONE1 and SUNE1 cells. Stable clones were selected using 0.5 μg/ml puromycin.

### Plasmid and small interfering RNA(siRNA) construction and transfection

The short hairpin RNA targeting LHX2 (sh-LHX2) (Supplementary Table 3) was synthesised and cloned into pLKO.1. siRNA targeting FGF1 (siFGF1) (Supplementary Table 3) was synthesised by RiboBio (Guangzhou, China). For LHX2 and FGF1 knockdown, HONE1 and SUNE1 cells were transfected with sh-LHX2 plasmids (4 μg) or siFGF1 (50 nM) and their corresponding control vectors with Lipofectamine 3000 Transfection Reagent (Invitrogen), according to the introduction, and harvested for assays 48 h after transfection.

### CCK-8 assay, clone-formation assay and EdU assay

For the CCK-8 assay, 2 × 10^3^ cells were placed into 96-well plates, incubated with CCK-8 reagent (Dojindo, Japan) and absorbance (450 nm) was measured every 24 h. For the colony-formation assay, 6 × 10^2^ cells were seeded into six-well plates, with new medium every 3 days. After 10 days, colonies were then fixed in methanol, stained with 0.1% crystal violet, and the colonies in each group were counted and the relative colonies were normalised to the control group. Following the standard EdU (5-ethynyl-2’-deoxyuridine) Assay protocol, tumour cells were added to EdU (Invitrogen, Cat. A10044, USA) as recommended dose and imaged by fluorescence microscopy.

### Wound-healing assay

Cells in six-well culture plates were cultured with serum-free medium for 12 h. A P200 pipette tip was used to scrape the cells in a straight line. The detached cells were gently washed with PBS twice and the remaining cells were cultured in serum-free medium. After incubation for 36 h, the width of the scratch was detected, and the migration rate in each group (migration rate = (original width of the wound width after cell migration)/original width of the wound).was quantified and normalised to the control group.

### Migration and invasion assays

Cells were digested and suspended in serum-free medium, and 3 × 10^4^ cells were placed into the upper 8-μm pore transwell chambers (Corning, NY, USA), which were coated with or without Matrigel (BD Biosciences, San Diego, CA, USA). Culture medium with 10% FBS was placed into the lower chamber to act as a chemoattractant. After incubation for 12 h or 24 h, cells were fixed in paraformaldehyde and stained with 0.1% crystal violet. Cells on the undersides of the filters were observed and counted under ×200 magnification. The relative migrated or invaded cells were normalised to the control group.

### Conditioned medium collection

HONE1 and SUNE1 cells were transfected with an FGF1 overexpression plasmid and the corresponding empty vector. After transfection (48 h), the medium was replaced, and the cells were cultured for another 48 h. The supernatant was collected and centrifuged to remove cell debris. The conditioned medium was used for further functional studies, including CCK-8, colony formation, wound healing and transwell migration and invasion assays. All experiments were conducted in triplicate.

### Immunofluorescence

Transfected cells cultured in 24-well plates were fixed and incubated with β-catenin primary antibodies (1:200, Proteintech, 17565-1-AP, Chicago, USA) overnight at 4 °C. After washing with PBS, fluorescent secondary antibodies (1:200; Proteintech, SA00013-2) were added to the cells and incubated at room temperature for 1 h. Images were captured after staining with DAPI solution.

### Xenograft tumour models and metastasis models

All animal research procedures were performed according to the detailed rules of the Animal Care and Use Ethics Committee of Guangzhou Medical University. Five-week-old female nude mice were purchased from Guangdong Medical Laboratory Animal Center (Guangzhou, China). Mice were divided into four groups at random (five mice in each group), and 2 × 10^6^ LHX2-overexpressed NPC cell lines and their control cell lines were injected into the dorsal flank of the mice. One week after injection, mice were treated with AZD4547 (12.5 mg/kg/d) or equal volume of vehicle every other day by oral gavage for 3 weeks. The volume of the tumour was detected every 3 days. The tumour weight and volume were monitored. Volume (mm^3^) = (L×W2)/2 (L: length, W: width). Animals were sacrificed 30 days later, and the tumours were excised for further examination.

For the tumour metastasis model, 1 × 10^6^ LHX2-overexpressed or Vector NPC cell lines were injected into the tail vein of nude mice (five mice in each group) and treated with AZD4547 (12.5 mg/kg/d) or equal volume of vehicle every other day by oral gavage. Animals were sacrificed 30 days later, lungs were removed, and hematoxylin eosin (H&E) staining was conducted.

### Luciferase reporter assay

The pGL3 luciferase reporter plasmids, containing wild-type or mutant promoter region of FGF1, were constructed. Transfected cells were co-transfected with the indicated luciferase reporters and *Renilla* luciferase reporter for 24 h. The cells were harvested, and the luciferase activity was detected using a Dual-Luciferase Reporter Assay Kit (Promega).

### Chromatin immunoprecipitation assay

The chromatin immunoprecipitation (ChIP) assays were performed as previously described [7]. In brief, LHX2 overexpression or control cells were prepared for cross-linking and sheared to 200–500 bp by sonication. The chromatin fraction was immunoprecipitated with anti-LHX2 antibody (1:50; Abcam, UK) or IgG (1:50; Abcam, UK; negative control). Real-time PCR assays were conducted to detect the enrichment of the FGF1 promoter occupancy. The sequences of the ChIP primers are shown in Supplementary Table 1.

### The gene set enrichment analysis

The Gene Set Enrichment Analysis (GSEA) software (version 2.0.13, ww.broadinstitute.org/gsea/) identified LHX2 expression-related pathways in GSE12452 and GSE53819 datasets (https://www.ncbi.nlm.nih.gov/geo/). In brief, the enrichment score for each gene set was calculated using the Kolmogorov–Smirnov statistic to identify the metastasis-related pathway and FGF associated with LHX2 expression.

### Statistical analysis

Statistical analysis was performed with SPSS 22.0 software (SPSS, Chicago, USA). Statistical analysis of the data was performed using the Student’s *t* test or analysis of variance (ANOVA). Spearman correlation analysis was applied to analyse the relationship between LHX2 and FGF1 mRNA expression in the Gene Expression Omnibus (GEO) datasets and the TCGA datasets. All tests were two-tailed; *P* <0.05 were considered statistically significant.

## Results

### LHX2 is highly expressed in NPC and correlated with poor clinical outcome

**Fig. 1:**
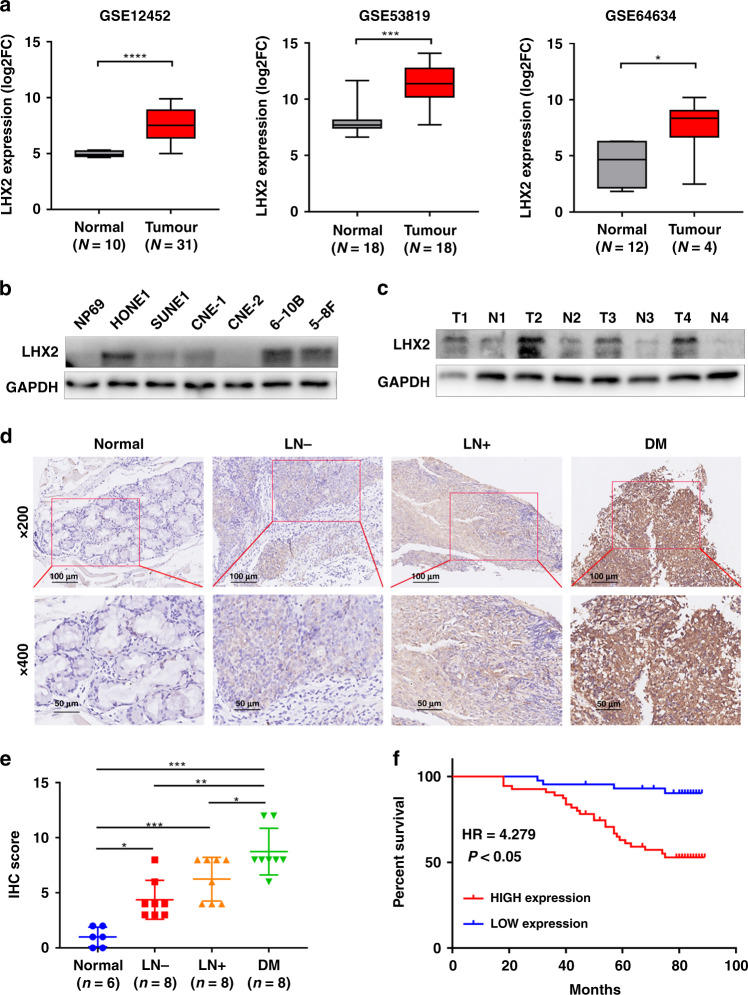
LHX2 is highly expressed in NPC and predicts poor survival. **a** Box plots showing LHX2 mRNA expression level is higher in nasopharyngeal carcinoma tissues than in normal tissues in GSE12452 (left), GSE53819 (middle) and GSE64634 (right). **b** Western blot analysis shows LHX2 expression level is higher in NPC cell lines than in normal nasopharyngeal epithelial cell lines. Each experiment was independently repeated at least three times. **c** Western blot analysis shows LHX2 expression level is higher in nasopharyngeal carcinoma tissues than in normal tissues. Each experiment was independently repeated at least three times. **d** Immunohistochemical staining and (**e**) statistical analysis of LHX2 in normal tissues, primary NPC tissues with (LN + ) or without (LN-) lymph node metastasis and primary NPC tissues with distant metastasis. **f** Kaplan–Meier survival curves indicate high expression of LHX2 is associated with poor survival in NPC patients. Data shown as mean ± SD. *P < 0.05, **P < 0.01, ***P < 0.001.

Genomic expression profiling of the TCGA database reveals LHX2 level is increased in many solid tumours (Supplementary Fig. S1A), and higher expression of LHX2 predicted poor survival in several solid tumours (Supplementary Fig. S1B), suggesting its potential oncogenic role in tumour development. To verify the expression of LHX2 in NPC, we analysed the expression level of LHX2 between NPC and normal tissues in three microarray-based high-throughput NPC datasets (GSE12452, GSE53819 and GSE64634). LHX2 mRNA expression was significantly upregulated in NPC clinical specimens (Fig. 1a). Next, we examined LHX2 expression in NPC cell lines and a normal nasopharyngeal epithelial cell line. Consistently, LHX2 protein level was significantly upregulated in NPC cell lines (Fig. 1b). Furthermore, western blot and IHC analysis showed LHX2 was dramatically upregulated in NPC tissues compared to normal tissues (Fig. 1c, d). Notably, LHX2 protein level was substantially higher in NPC tissues with regional lymph node and distant metastasis than in tissues without metastasis (Fig. 1e). Moreover, the LHX2 level was higher in NPC tissues of the T3/T4 stage than T1/T2 stage (Supplementary Fig. S2). By analysing the relationship between LHX2 expression and clinical data in a cohort of 98 paraffin-embedded NPC biopsy samples, we found a higher LHX2 level was significantly associated with T, N and TNM staging (*P* < 0.05, Table 1). Moreover, Kaplan–Meier survival analysis demonstrated that patients with higher LHX2 expression have significantly worse overall survival (*P* < 0.05, Fig. 1f). Taken together, these data suggest that LHX2 is upregulated in NPC and associated with poor survival.

**Table 1:** Clinical characteristics of NPC patients with either low or high LHX2 expression.

Characteristics	No. of patients	Expression of *LHX2*		*P* value
		Low, *n* (%)	High, *n* (%)	
Age				
≤45	35	13 (30.2)	22 (62.9)	0.317
>45	63	30 (47.6)	33 (52.3)	
Sex				
Male	74	34 (45.9)	40 (54.1)	0.469
Female	24	9 (37.5)	15 (62.5)	
T stage				
T1–T2	47	1 (55.3)	21 (44.7)	**0.028**
T3–T4	51	17 (33.3)	34 (66.7)	
N stage				
N0	28	12 (66.7)	6 (33.3)	**0.031**
N1–N3	70	31 (38.7)	49 (61.3)	
TNM stage				
III–IV	84	33 (39.3)	51 (60.7)	**0.025**
I–II	14	10 (71.4)	4 (28.6)	
Distant metastasis				
Yes	6	1 (16.7)	5 (83.3)	0.166
No	92	42 (45.7)	50 (54.3)	
Death				
Yes	29	4 (13.8)	25 (82.6)	**0.000**
No	69	39 (56.5)	30 (43.5)	

Bold values indicate statistical significance *p* < 0.05.

### LHX2 promotes NPC cells growth both in vitro and in vivo

**Fig. 2:**
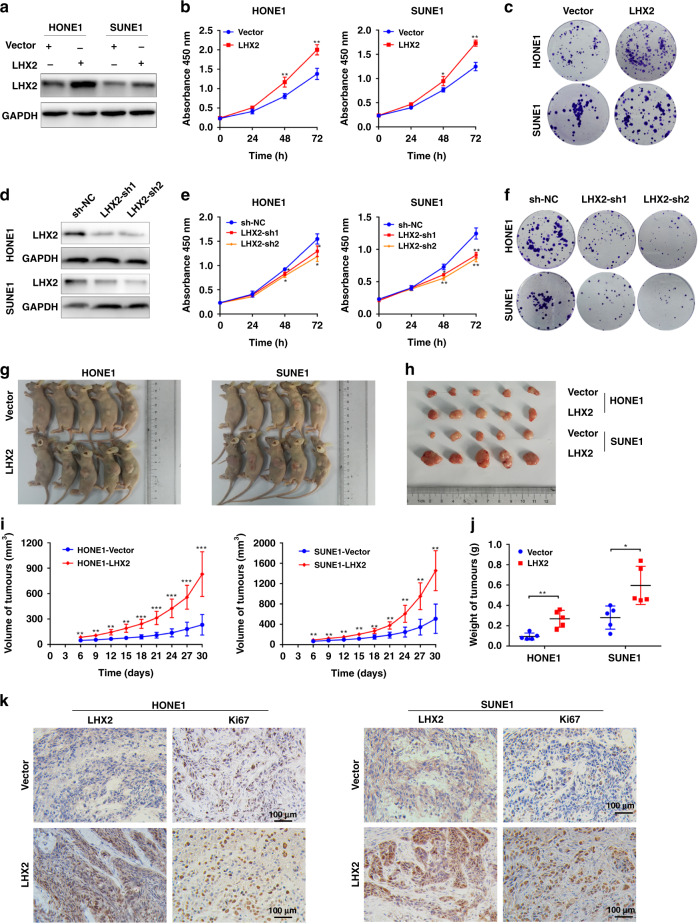
LHX2 promotes NPC cell growth in vitro and in vivo. **a** Western blot analysis of LHX2 protein expression in HONE1 and SUNE1 cells stably overexpressing LHX2. Each experiment was independently repeated at least three times. **b** The CCK-8 assay and **c** the colony-formation assay of HONE1 and SUNE1 cells stably overexpressing LHX2. Each experiment was independently repeated at least three times. **d** Western blot analysis of LHX2 protein expression in HONE1 and SUNE1 cells transfected with LHX2 shRNAs or control cells. Each experiment was independently repeated at least three times. **e** The CCK-8 assay and **f** the colony-formation assay of HONE1 and SUNE1 cells transfected with LHX2 shRNAs or control. Each experiment was independently repeated at least three times. **g**, **h** Images of Xenograft tumours of nude mice 30 days after injecting with HONE1 and SUNE1 cells stably overexpressing LHX2 or vector (*n* = 5 in each group). **i** Tumour volume growth curves. **j** Average xenograft tumour weights. **k** Immunohistochemistry assay of LHX2 and Ki67 protein expression in xenograft tumours. Data shown as mean ± SD. *P < 0.05, **P < 0.01, ***P < 0.001.

To evaluate the effect of LHX2 on NPC cell growth, HONE1 and SUNE1 were transduced with lentivirus expressing LHX2 (Fig. 2a and Supplementary Fig. S3A). The CCK-8 and colony-formation assays demonstrated that NPC cells overexpressing LHX2 achieved significantly faster viability (Fig. 2b, c and Supplementary Fig. S3B). Similarly, 5-ethynyl-2’-deoxyuridine (EdU) assays also indicated LHX2 promotes the proliferation of HONE1 and SUNE1 cells (Supplementary Fig. S3C). In contrast, LHX2 inhibition decreased NPC cell proliferation (Fig. 2d–f and Supplementary Fig. S3D–F).

To further explore the roles of LHX2 on NPC growth in vivo, xenograft tumour models were established. Tumour growth promotion was observed in LHX2-overexpressing cells compared to control cells (Fig. 2g–i). In parallel, the average weight of tumours was significantly higher in LHX2-overexpressed tumours than in control tumours (Fig. 2j). IHC analysis of xenograft tissues showed an increased expression of cell proliferation marker Ki67 in LHX2-overexpressing groups compared with control groups (Fig. 2k). Collectively, these findings indicate that LHX2 promotes NPC cell growth both in vitro and in vivo.

### LHX2 promotes NPC cells migration, invasion and EMT

**Fig. 3:**
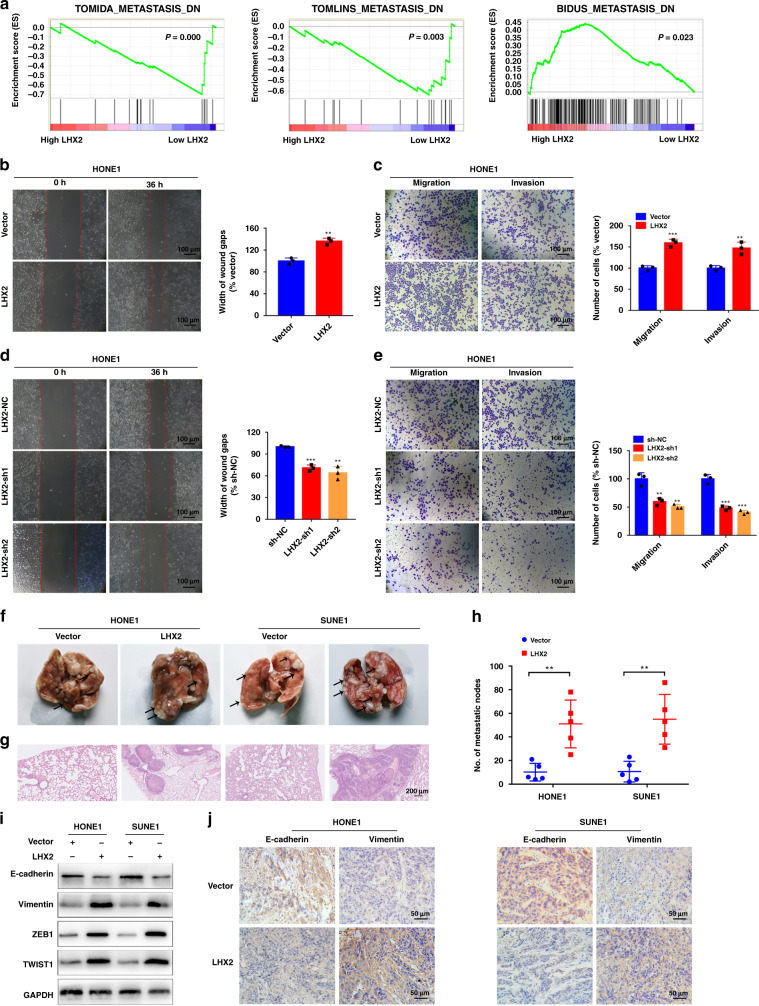
LHX2 strengthens NPC cells migration, invasion and metastasis. **a** GSEA enrichment plots demonstrated that metastasis was associated with upregulation of LHX2 in GSE12452 (left and middle) and GSE53819 (right). **b** Wound healing and (**c**) transwell migration and invasion assays in HONE1 cells stably overexpressing LHX2 or empty vector. Each experiment was independently repeated at least three times. **d** Wound healing and (**e**) transwell migration and invasion assays in HONE1 cells transfected with LHX2 shRNAs or control. Each experiment was independently repeated at least three times. **f**–**h** Representative images of metastatic lungs (**f**), representative HE images (**g**) and numbers of metastatic foci per lung (**h**) injected with HONE1 and SUNE1 cells stably overexpressing LHX2 or empty vector (*n* = 5 in each group). **i** Western blot analysis of EMT-related protein expression in HONE1 and SUNE1 cells stably overexpressing LHX2 or empty vector. Each experiment was independently repeated at least three times. **j** IHC analysis of E-cadherin and vimentin protein expression in xenograft tumours. Data shown as mean ± SD. *P < 0.05, **P < 0.01, ***P < 0.001.

GSEA based on the GSE12452 and GSE53819 databases revealed that LHX2 was associated with NPC metastasis (Fig. 3a). Wound-healing and transwell assays indicated overexpression of LHX2 significantly promoted the migration and invasion of NPC cells (Fig. 3b, c and Supplementary Fig. S4A, B). However, inhibition of LHX2 suppressed the migratory and invasive abilities of NPC cells (Fig. 3d, e and Supplementary Fig. S4C, D). Moreover, we explored the role of LHX2 in tumour metastasis in vivo via a lung metastasis model. The results showed ectopic LHX2 overexpression significantly increased the number of metastatic nodules, as confirmed by stained lung sections (Fig. 3f–h).

EMT is a crucial process for tumour metastasis. We examined whether LHX2 was associated with EMT in NPC. Western blot analysis showed that LHX2 overexpression decreased the expression of E-cadherin and increased the expression of vimentin, Zinc Finger E-Box Binding Homeobox 1 (ZEB1) and Twist Family BHLH Transcription Factor 1 (TWEST1) (Fig. 3i). In addition, a decreased level of E-cadherin and an increased level of Vimentin were determined in LHX2-overexpressing tumours (Fig. 3j). These results reveal that LHX2 promotes cell migration, invasion, metastasis and EMT in NPC cells.

### LHX2 activates pro-survival and β-catenin signalling pathways through transcriptionally regulating FGF1 expression

**Fig. 4:**
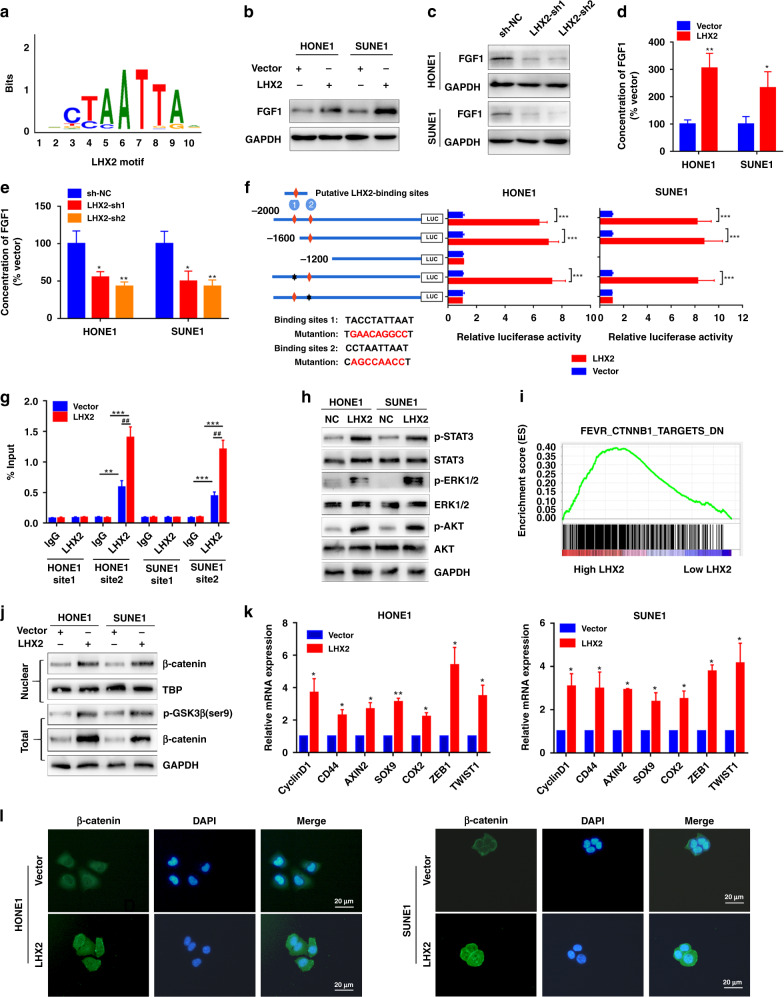
LHX2 activates pro-survival and β-catenin signalling pathways via transcriptionally regulating FGF1 expression. **a** LHX2 motif. **b**, **c** Western blot analysis of FGF1 expression in HONE1 and SUNE1 cells with LHX2 overexpression (**b**) or knockdown (**c**). Each experiment was independently repeated at least three times. **d**, **e** ELISA assay of FGF1 expression in the supernatant derived from HONE1 and SUNE1 cells with LHX2 overexpression (**d**) or knockdown (**e**). Each experiment was independently repeated at least three times. **f** Luciferase activity of the reporter gene driven by serially truncated/mutated FGF1 promoters. Each experiment was independently repeated at least three times. **g** ChIP assay confirms the direct binding of LHX2 to the FGF1 promoters in HONE1 and SUNE1 cells. Each experiment was independently repeated at least three times. **h** Western blot assay reveals the p-STAT3, pERK1/2 and p-AKT were activated by LHX2. Each experiment was independently repeated at least three times. **i** GSEA enrichment plots demonstrates that β-catenin signalling is associated with upregulation of LHX2 in GSE53819. **j** Western blot assay of β-catenin and Ser9-GSK3β in HONE1 and SUNE1 cells stably overexpressing LHX2 or containing the empty vector. Each experiment was independently repeated at least three times. **k** RT-qPCR analysis of β-catenin response genes in HONE1 and SUNE1 cells stably overexpressing LHX2 or containing the empty vector. Each experiment was independently repeated at least three times. **l** Representative images of immunofluorescent staining of β-catenin in HONE1 and SUNE1 cells stably overexpressing LHX2 or containing the empty vector. Each experiment was independently repeated at least three times. Data shown as mean ± SD. *P < 0.05, **P < 0.01, ***P < 0.001.

Considering LHX2 is a transcription factor, we referred to the accessible online TF ChIP-seq database (http://dc2.cistrome.org/) to explore its potential downstream targets. We found FGF1 was a putative target of LHX2 that LHX2 motif (Fig. 4a) could bind to its promoter. Consistently, overexpression of LHX2 increased FGF1 expression (protein and mRNA) while inhibition of LHX2 could reduce FGF1 expression (Fig. 4b, c and Supplementary Fig. S5A, B). Furthermore, ELIAS assay showed overexpression of LHX2 increased FGF1 expression in the supernatant, while the reverse was observed in LHX2-silenced NPC cells (Fig. 4d, e). FGF1 also has higher mRNA expression in NPC clinical specimens (Supplementary Fig. S5C). To confirm the LHX2-binding sites on FGF1, deletions and selective mutations were generated into the FGF1 promoter sequences. We identified that the FGF1 promoter binding site 2 was the putative LHX2-binding site (Fig. 4f), and ChIP real-time PCR analysis confirmed the affinity of LHX2 to the FGF1 promoter (Fig. 4g).

Accumulating evidence has revealed that FGF1 can bind to FGFR and activate the STAT3, AKT and ERK signalling pathways [28]. Therefore, we detected these signalling pathways and found LHX2 overexpression activated p-STAT3, p-ERK and p-AKT (Fig. 4h). In light of the activation of p-AKT by LHX2, we hypothesised that LHX2 may facilitate β-catenin stability via downregulating glycogen synthase kinase-3β (GSK3β) activity by phosphorylation at Ser9 of GSK3β. GSEA demonstrated that LHX2 was associated with the β-catenin signalling pathway (Fig. 4i). LHX2 overexpression increased nuclear levels of β-catenin and total expression levels of β-catenin and p-Ser9-GSK3β in both HONE1 and SUNE1 cells (Fig. 4j). Moreover, the expression levels of β-catenin-targeted genes were substantially increased in LHX2-overexpressed NPC cells, including ZEB1 and TWST1 (Fig. 4k). Immunofluorescence analysis showed that LHX2 overexpression induced the accumulation of nuclear β-catenin (Fig. 4l). Importantly, FGF1 expression was positively associated with ZEB1 and TWIST1 expression in pan-cancers (Supplementary Figs. S6 and S7). These findings suggest that LHX2 activates pro-survival, β-catenin signalling pathways via transcriptionally regulating FGF1 expression in NPC.

### FGF1 mediates LHX2-induced proliferation, migration and invasion in an autocrine or paracrine manner

**Fig. 5:**
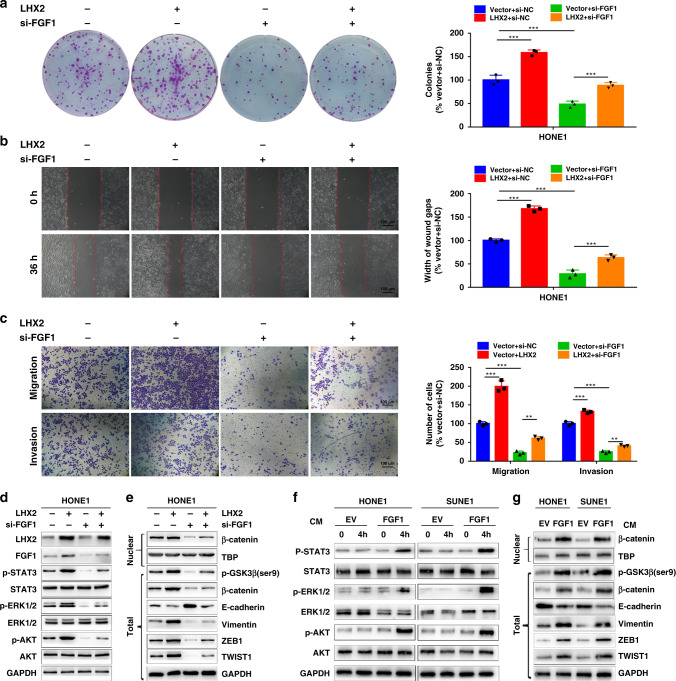
FGF1 mediates LHX2-induced proliferation, migration and invasion in an autocrine or paracrine manner. **a**–**e** FGF1 siRNA was transfected into HONE1 cells stably overexpressing LHX2 or the empty vector. Colony-formation assay (**a**), wound-healing assay (**b**) and transwell migration and invasion assays (**c**). Western blot analysis of LHX2, FGF1 and pro-survival signalling pathways (**d**). Western blot analysis of β-catenin signalling pathway and EMT-related proteins (**e**). Each experiment was independently repeated at least three times. **f**, **g** HONE1 and SUNE1 cells were treated with FGF1-CM or EV-CM for the indicated time. Western blot analysis of pro-survival signalling pathways (**f**). Western blot analysis of β-catenin signalling pathway and EMT-related proteins (**g**). Each experiment was independently repeated at least three times. Data shown as mean ± SD. *P < 0.05, **P < 0.01. FGF1-CM FGF1-condition medium, EV empty vector.

To explore whether FGF1 contributes to LHX2-induced proliferation, migration and invasion in NPC cells. We inhibited FGF1 expression in NPC cells overexpressing LHX2 with siRNA. We found FGF1 inhibition restrained the proliferative, migratory and invasive abilities of NPC cells induced by ectopic expression of LHX2 (Fig. 5a–c and Supplementary Fig. S8A–C). Furthermore, inhibition of FGF1 significantly blocked the activation of ERK1/2, AKT, STAT3 and β-catenin signalling pathways stimulated by LHX2 overexpression (Fig. 5d, e). As cancer cells secrete FGFs, we transfected NPC cells with an FGF1 overexpression plasmid or empty vector to determine whether FGF1 promotes NPC progression in an autocrine/paracrine secretion manner. The conditioned medium (CM) derived from transfected HONE1 and SUNE1 cells was centrifuged and added to the indicated NPC cells (Supplementary Fig. S8D). Notably, FGF1-CM activated STAT3, ERK and AKT signalling as shown by the consistently elevated expression levels of p-STAT3, pERK1/2 and p-AKT when LHX2 was overexpressed (Fig. 5f). Moreover, Ser9-GSK3β/β-catenin signalling and EMT-related proteins were also stimulated by FGF1-CM (Fig. 5g). These observations reveal that FGF1 serves as a functional target of LHX2 in NPC cells and inhibition of FGF1 could block the LHX2-induced proliferation, migration and invasion of NPC cells.

### Blocking FGF1/FGFR signalling suppresses the growth and metastasis of NPC cells

**Fig. 6:**
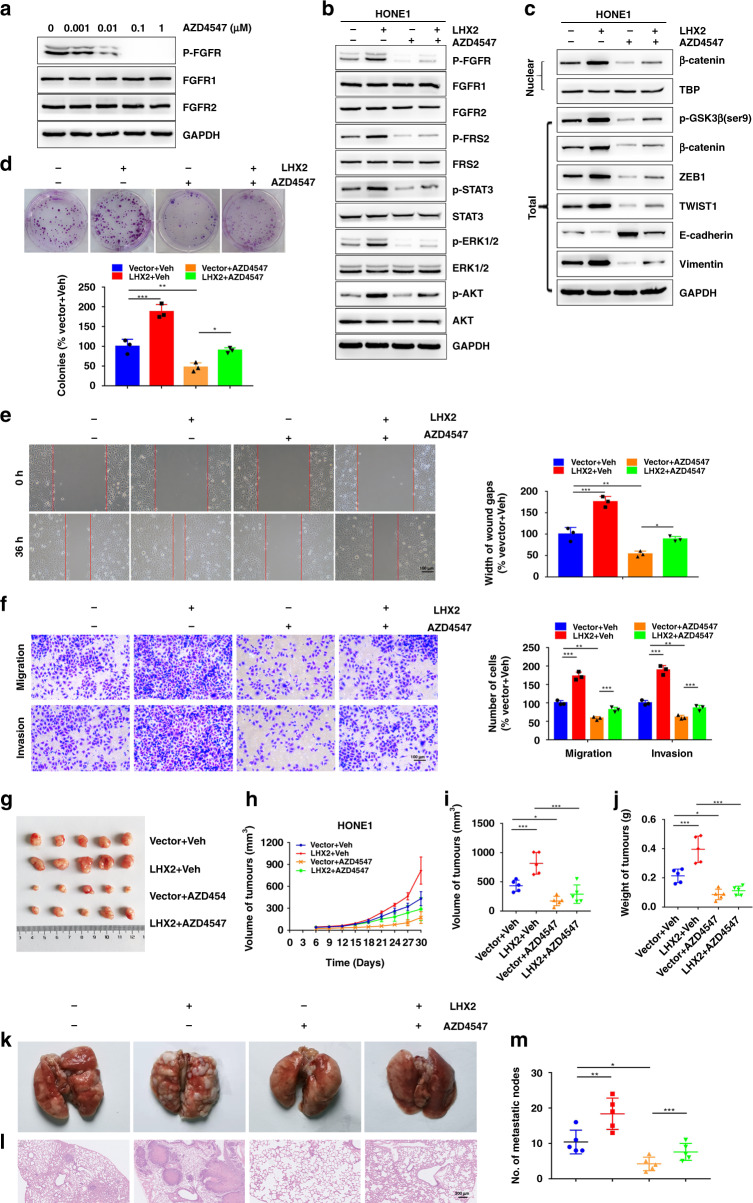
Blocking FGF1/FGFR signalling suppresses the proliferation, migration and invasion of NPC cells. **a** HONE1 cells were incubated for 4 h of AZD4547 at different concentrations and then lysed and immunoblotted for the indicated proteins. **b**, **c** HONE1 cells stably overexpressing LHX2 or the empty vector were treated with AZD4547 (100 nM) or DMSO. Whole-cell lysates were subjected to western blot analysis. Each experiment was independently repeated at least three times. **d**–**f** HONE1 cells stably overexpressing LHX2 or the empty vector were treated with AZD4547 (100 nM) or DMSO for 48 h. The cells were harvested and subjected to (**d**) colony-formation assay, (**e**) wound-healing assay and (**f**) transwell migration and invasion assays. Each experiment was independently repeated at least three times. **g**–**j** LHX2-overexpressed or vector HONE1 cells (2 × 10^6^) were injected into the dorsal flank of mice. One week after injection, the mice were treated with AZD4547 (12.5 mg/kg/d) or vehicle orally for 3 weeks. Images of Xenograft tumours (**g**). Tumour volume growth curves (**h**). The volume of xenograft tumours (**i**). Average xenograft tumour weights (**j**). LHX2-overexpressed or vector HONE1 cells (1 × 10^6^) were injected into the tail vein of nude mice and the mice were treated with AZD4547 (12.5 mg/kg/d) or vehicle orally for 3 weeks. **k**–**m** Representative images of metastatic lungs (**k**), representative HE images (**l**) and numbers of metastatic foci per lung (**m**) (*n* = 5 in each group). Data are shown as means ± SD. *P < 0.05, **P < 0.01, ***P < 0.001.

FGF1 commonly stimulates downstream signalling cascades by phosphorylating the FGFR [23]. To assess the impact exerted by inhibiting the FGF/FGFR axis on NPC cell proliferation, migration and invasion, NPC cells were treated with AZD4547, an FGFR-specific inhibitor. We observed extracellular recombinant FGF1 caused the activation of FGFR, and thus stimulated the cascade of phosphorylation of downstream signalling pathways, which could be abrogated by AZD4547 (Supplementary Fig. S9A, B). Consistently, administration of FGF1 dramatically increased the cell proliferation as well as migration and invasion of HONE1 cells, which could be attenuated by AZD4547 (Supplementary Fig. S9C–F). To get further insights into the impact of FGF/FGFR signalling blockage on LHX2-induced proliferation, migration and invasion in NPC, we administrated NPC cells with AZD4547. The inhibition of cellular FGFR phosphorylation was observed in a dose-dependent manner by AZD4547 (Fig. 6a and Supplementary Fig. S10A). In addition, the phosphorylation of FRS2, downstream marker of FGFR signalling, LHX2-mediated induction of STAT3, ERK, AKT and β-catenin signalling as well as EMT were also significantly abrogated by AZD4547 (Fig. 6b, c). Consistently, administration of AZD4547 dramatically blocked the positive effect of LHX2 on HONE1 cell proliferation, migration and invasion in vitro (Fig. 6d–f and Supplementary Fig. S10B). Furthermore, AZD4547 treatment significantly revised the accelerated tumour growth and metastasis by LHX2 overexpression in vivo (Fig. 6g–m). Together, these findings revealed an oncogenic role of the LHX2-induced FGF/FGFR signalling pathway in NPC tumorigenesis and the FGF/FGFR trapping by AZD4547 could reverse these effects.

### The expression of LHX2 and FGF1 are positively correlated in NPC tissues

**Fig. 7:**
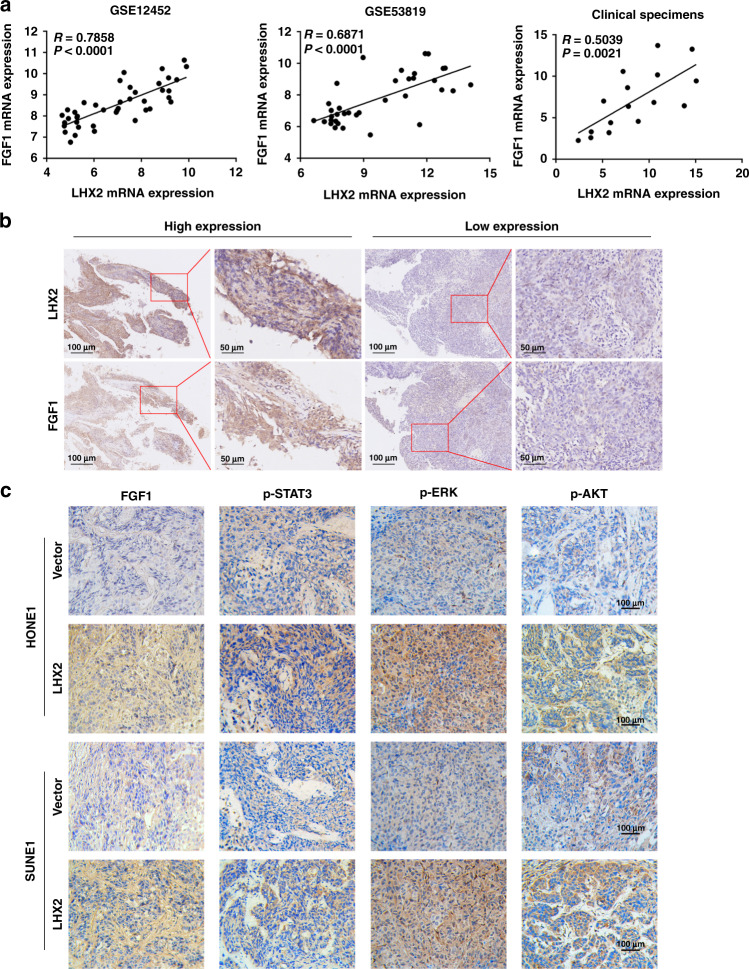
LHX2 is positively correlated with FGF1 in NPC. **a** Correlations between LHX2 and FGF1 mRNA expression in NPC tissues. **b** Representative immunostaining of LHX2 and FGF1 in consecutive paraffin sections of NPC tissues. **c** Representative immunostaining of FGF1, p-STAT3, p-ERK, p-AKT in LHX2 overexpression and control xenograft tumours.

To extend our findings in human NPC tissues, we investigated the relationship between LHX2 and FGF1 expression in NPC tissues. Pearson correlation analysis showed that FGF1 and LHX2 mRNA expression were positively correlated in NPC clinical specimens (Fig. 7a). Consistently, TCGA datasets from other types of cancers further verified the positive correlation between FGF1 and LHX2 expression (Supplementary Fig. S11). Moreover, a positive correlation between FGF1 and LHX2 protein expression was confirmed in NPC tissue (Fig. 7b). In addition, high expression of FGF1, p-STAT3, p-ERK and p-AKT were observed in LHX2-overexpressing xenografts, as shown by IHC analysis (Fig. 7c).

## Discussion

**Fig. 8:**
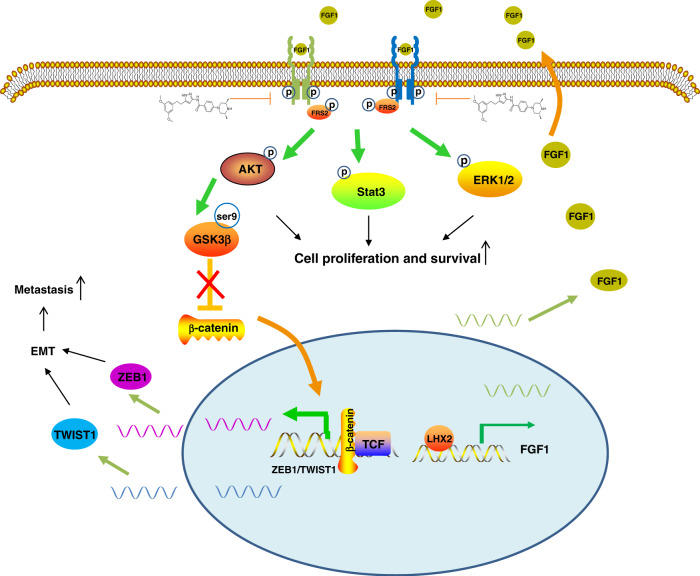
A working model of LHX2-induced NPC progression. LHX2 transcriptionally activates FGF1 expression. Tumour cells produce FGF1, which binds to FGFR, and the subsequent downstream signalling occurs through the intracellular receptor substrates FGFR substrate 2 (FRS2), resulting in the phosphorylation and activation of MAPK/ERK, JAK2/STAT3, and PI3K/AKT signalling pathways. (i) These pro-survival pathways are responsible for the promotion of tumour proliferation and growth. (ii) Phosphorylated AKT stimulates the Ser9-GSK3β/β-catenin signalling, leading to the EMT of NPC via the β-catenin targeted ZEB1 and TWIST1 genes and promotes tumour cell migration and invasion. (iii) FGF/FGFR trapping by AZD4547 could block the LHX2/FGF1-induced promotion on tumour growth and metastasis.

This study identified LHX2 as a novel transcription factor related to NPC progression. LHX2 transcriptionally regulated FGF1 expression and promoted the growth and metastasis of NPC in an FGF1/FGFR-dependent manner. Furthermore, FGF/FGFR trapping by AZD4547 could block the LHX2/FGF1-induced promotion of tumour growth and metastasis. Our study suggests LHX2 as a novel biomarker for NPC diagnosis and prognosis evaluation, as well as targets for therapeutic treatment (Fig. 8).

A precise method for measuring the risk of recurrence or distant metastasis in NPC patients is needed in the clinic. By applying bioinformatic analysis of the TCGA and GEO databases, we found that LHX2 expression was dramatically increased in some solid tumours, including NPC. The carcinogenesis of LHX2 has been confirmed in osteosarcoma and prostate cancer [29, 30]. However, the role and mechanism of LHX2 in NPC were largely unknown. In this study, we verified that LHX2 was upregulated in NPC and high expression of LHX2 in NPC patients had worse clinical outcomes. These results suggest LHX2 is a promising prognostic biomarker for NPC detection and therapy.

FGFs activate MAPK-ERK, JAK2/STAT3 and PI3K/AKT signalling pathways, which has been identified as key mediators in tumour progression promotion [31, 32]. FGF1 was reported to promote the proliferation and invasion of LCSCs dependent on the MAPK/ERK signalling pathway [21] SH2B1β plays an important role in neurite outgrowth via enhancing and prolonging FGF1-induced MEK-ERK1/2 and PI3K-AKT pathways [33]. FGFR activation induces the accumulation of hyaluronan (HA) and increases the proliferation, migration and therapeutic resistance of breast cancer cells. Importantly, FGFR-mediated HA accumulation requires downstream activation of the STAT3 signalling pathway [34]. Consistent with these observations, we found FGF1 promote the growth of NPC cells primarily via activating phosphorylation of STAT3, ERK1/2 and AKT, which could be blocked by siFGF1 or FGFR inhibitor.

EMT is a physiological phenomenon, during this process, epithelial cells possess the characteristics of mesenchymal cells, including enhanced motility and invasive ability [35]. As the key initial step in the EMT, the downregulation of E-cadherin expression was observed in metastatic tumours, the transcriptional level of E-cadherin is repressed by several factors including, ZEB1 and Twist1, which have been identified as the effectors of β-catenin/TCF4 signalling in tumour invasiveness [36–38]. LHX2 has been reported to play an important role in epithelial–mesenchymal interactions, as identified in hair, liver and brain [10, 39, 40]. In this study, we demonstrated that LHX2 promotes EMT of NPC cells via transcriptionally activating FGF1, upregulating ZEB1 and TWIST1 through the β-catenin signalling pathway. In the absence of Wnt signal, APC/Axin/GSK3β could target β-catenin leading to its ubiquitination and proteasomal degradation [41]. The activation of GSK3β requires phosphorylation at Tyr216, but many signalling pathways are reported to downregulate GSK3β activity through phosphorylation at Ser9 [42, 43]. We found FGF1 binds to the FGFR, resulting in the phosphorylation of Akt, which in turn phosphorylates GSK3β at Ser9, rendering the kinase-inactive and resulting in decreased phosphorylation of downstream substrates, including β-catenin.

Although Tian-Song Liang has found that MicroRNA-506 can inhibit NPC tumour growth and metastasis by downregulating LHX2 [44], our study further extended the downstream targets and signalling pathway by which LHX2 serve as the oncogene in NPC, providing potential therapeutic strategies, such as FGF1/FGFR inhibitors. Treatment of FGF1 siRNA or FGFR inhibitor, efficiently decreased the growth and metastasis in LHX2-overexpressing NPC cells. The relevance of autocrine/paracrine FGF signalling has been shown in the development of various tumours, including multiple myeloma [45], breast cancer [46] and uveal melanoma [47], suggesting a promising target for tumour therapy. NSC12, a TX3-derived small molecule, is able to suppress FGFR activation and tumour growth in various FGF-dependent cancer models [48, 49]. In a Phase II study, erdafitinib, a tyrosine kinase inhibitor of FGFR1–4, exhibits antitumor activity in advanced urothelial carcinoma with FGFR alterations [50]. AZD4547 is a novel selective inhibitor of the FGFR1/2 tyrosine kinases. Administration of AZD4547 resulted in potent dose-dependent antitumor activity in preclinical models and has reached Phase II clinical investigations in malignancies with FGFR alterations, including lung cancer, endometrial cancer, multiple myeloma and breast cancer [51, 52]. Although AZD4547 did not meet the primary endpoint of the study, it did show a modest benefit in patients with FGFR mutations and fusions. Moreover, different approaches to targeting the FGF/FGFR system have been verified [47] and various selective TKI FGFR inhibitors have reached clinical trials on different kinds of tumours [53]. These studies highlight the potential of FGFR inhibitors as cancer therapeutics.

In summary, this study demonstrated that LHX2 is frequently upregulated in NPC and promotes NPC cell growth and metastasis both in vitro and in vivo. Furthermore, the role of LHX2 in NPC may depend on its transcriptional promotion of FGF1 and activated FGF1/FGFR signalling. Overall, our findings provide a new insight into the potential mechanism by which LHX2 regulates FGF1 expression in NPC progression.

## Supplementary information

Supplementary data

checklist

## Data Availability

The obtained results of the research are available on reasonable request.
