# Supplementary material for: LHX2 facilitates the progression of nasopharyngeal carcinoma via activation of the FGF1/FGFR axis
Source: Br J Cancer. 2022 Jul 21;127(7):1239–53. doi: 10.1038/s41416-022-01902-7 (PMC9519904; doi:10.1038/s41416-022-01902-7)
Supplement: Supplementary file 1 — Supplementary data [file 41416_2022_1902_MOESM1_ESM.docx]

##### LHX2 facilitates the progression of nasopharyngeal carcinoma via activating the FGF1/FGFR axis

**Supplementary Table 1. Sequences of ChIP primers**

| SBE1 | Forward(5’-3’) | GGGCAACAGAGCGAGACT |
| --- | --- | --- |
|  | Reverse(5’-3’) | AGTGCAATGGTGCAATGTTGGCTCA |
| SBE2 | Forward(5’-3’) | AGTTTGGGGACTTTGATA |
|  | Reverse(5’-3’) | TTTTTTAATTCAACAACTTTGCTT |

**Supplementary Table 2. Sequences of PCR primers used in this study**

| LHX2 | Forward(5’-3’) | ACGCCAAGGACTTGAAGCAGCT |
| --- | --- | --- |
|  | Reverse(5’-3’) | TTTCCTGCCGTAAGAGGTTGCG |
| FGF1 | Forward(5’-3’) | CATGCCAGGTTAGGAAGGCA |
|  | Reverse(5’-3’) | ATGGTATCCCCTCAGCCAGT |
| cyclinD1 | Forward(5’-3’) | GCTGCGAAGTGGAAACCATC |
|  | Reverse(5’-3’) | CCTCCTTCTGCACACATTTGAA |
| CD44 | Forward(5’-3’) | CTGCCGCTTTGCAGGTGTA |
|  | Reverse(5’-3’) | CATTGTGGGCAAGGTGCTATT |
| AXIN2 | Forward(5’-3’) | CTGGCTCCAGAAGATCACAAAG |
|  | Reverse(5’-3’) | CATCCTCCCAGATCTCCTCAAA |
| SOX9 | Forward(5’-3’) | AGCGAACGCACATCAAGAC |
|  | Reverse(5’-3’) | CTGTAGGCGATCTGTTGGGG |
| COX2 | Forward(5’-3’) | CTGGCGCTCAGCCATACAG |
|  | Reverse(5’-3’) | CGCACTTATACTGGTCAAATCCC |
| ZEB1 | Forward(5’-3’) | GATGATGAATGCGAGTCAGATGC |
|  | Reverse(5’-3’) | ACAGCAGTGTCTTGTTGTTGT |
| TWIST1 | Forward(5’-3’) | GTCCGCAGTCTTACGAGGAG |
|  | Reverse(5’-3’) | GCTTGAGGGTCTGAATCTTGCT |

**Supplementary Table 3. Sequences of shRNA Against Specific Targets**

| sh-LHX2-1 | 5’-3’ | GCAACCTCTTACGGCAGGAAA |
| --- | --- | --- |
| sh-LHX2-2 | 5’-3’ | GTGCACCACGTGTAACAAGAT |
| si-FGF1 | 5’-3’ | TGCAAAAGTGGGGCTAAATGAAG |

**Supplementary Figures**

**Fig.S1**

**
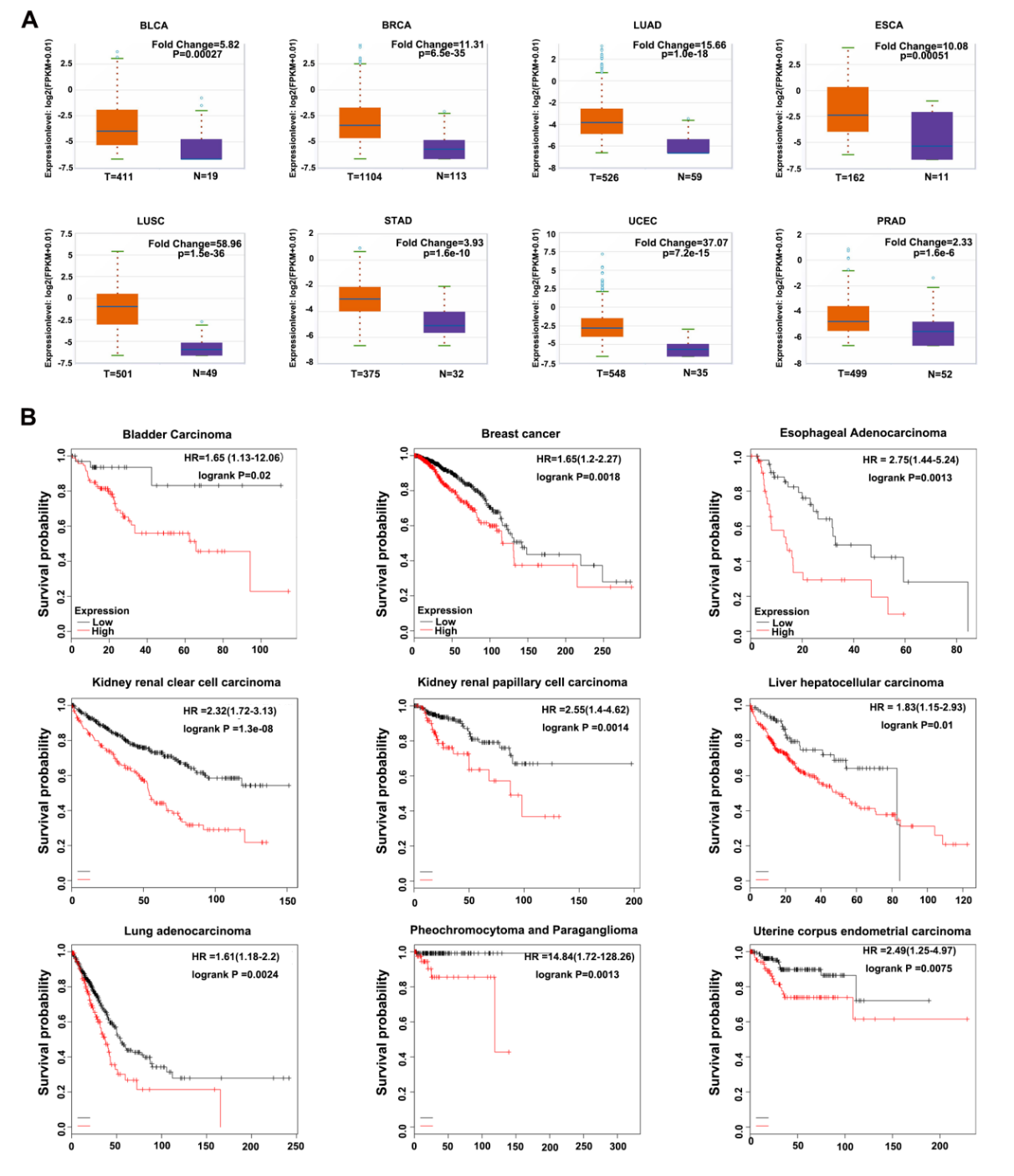
**

**Figure S1. LHX2 is upregulated in solid tumors and predicts poor prognosis**.

(A) LHX2 mRNA expression analysis in bladder urothelial carcinoma (BLCA), breast invasive carcinoma (BRCA), lung adenocarcinoma (LUAD), cervical squamous cell carcinoma and endocervical adenocarcinoma (ECSA), lung squamous cell carcinoma (LUSC), stomach adenocarcinoma (STAD), uterine corpus endometrial carcinoma (UCEC) and prostate adenocarcinoma (PRAD). Data were analyzed using starbase online software (https://starbase.sysu.edu.cn/). (B) Kaplan–Meier analysis of overall survival for patients with bladder carcinoma, breast cancer, esophageal adenocarcinoma, kidney renal clear cell carcinoma, kidney renal papillary cell carcinoma, liver hepatocellular carcinoma, Lung adenocarcinoma, pheochromocytoma and paraganglioma, uterine corpus, and endometrial carcinoma, stratified by low and high expression of LHX2. HR, hazard ratio.

**Fig. S2**

**
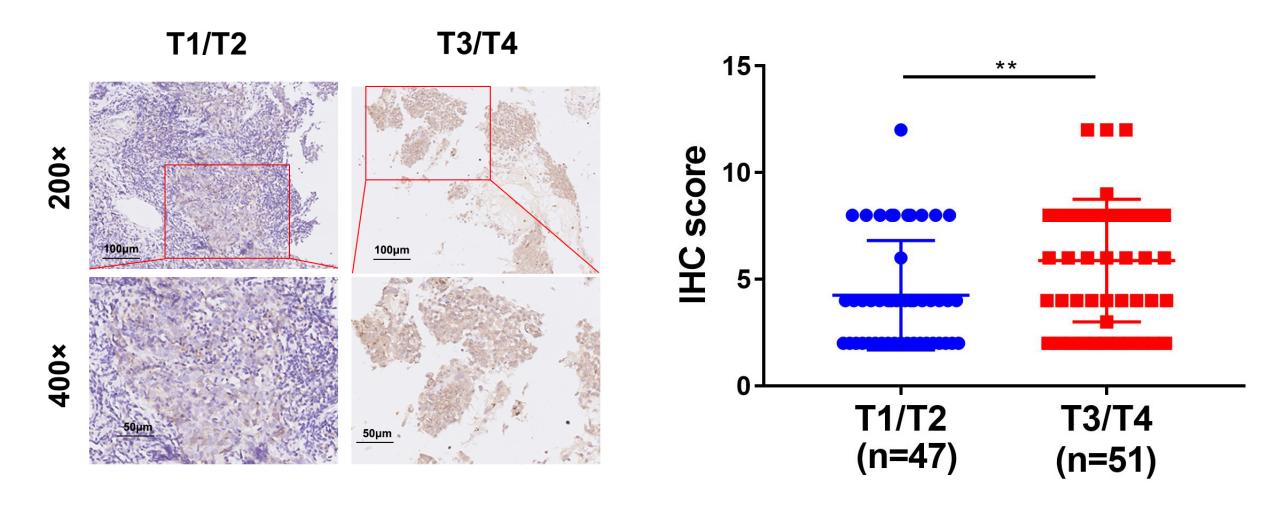
**

**Figure S2. LHX2 level is significantly associated with T stage.** (A) Immunohistochemical staining and (B) statistical analysis of LHX2 in NPC tissues in T1/T2 and T3/T4. Data shown as mean ± SD. ***p* < 0.01.

**Fig. S3**

**
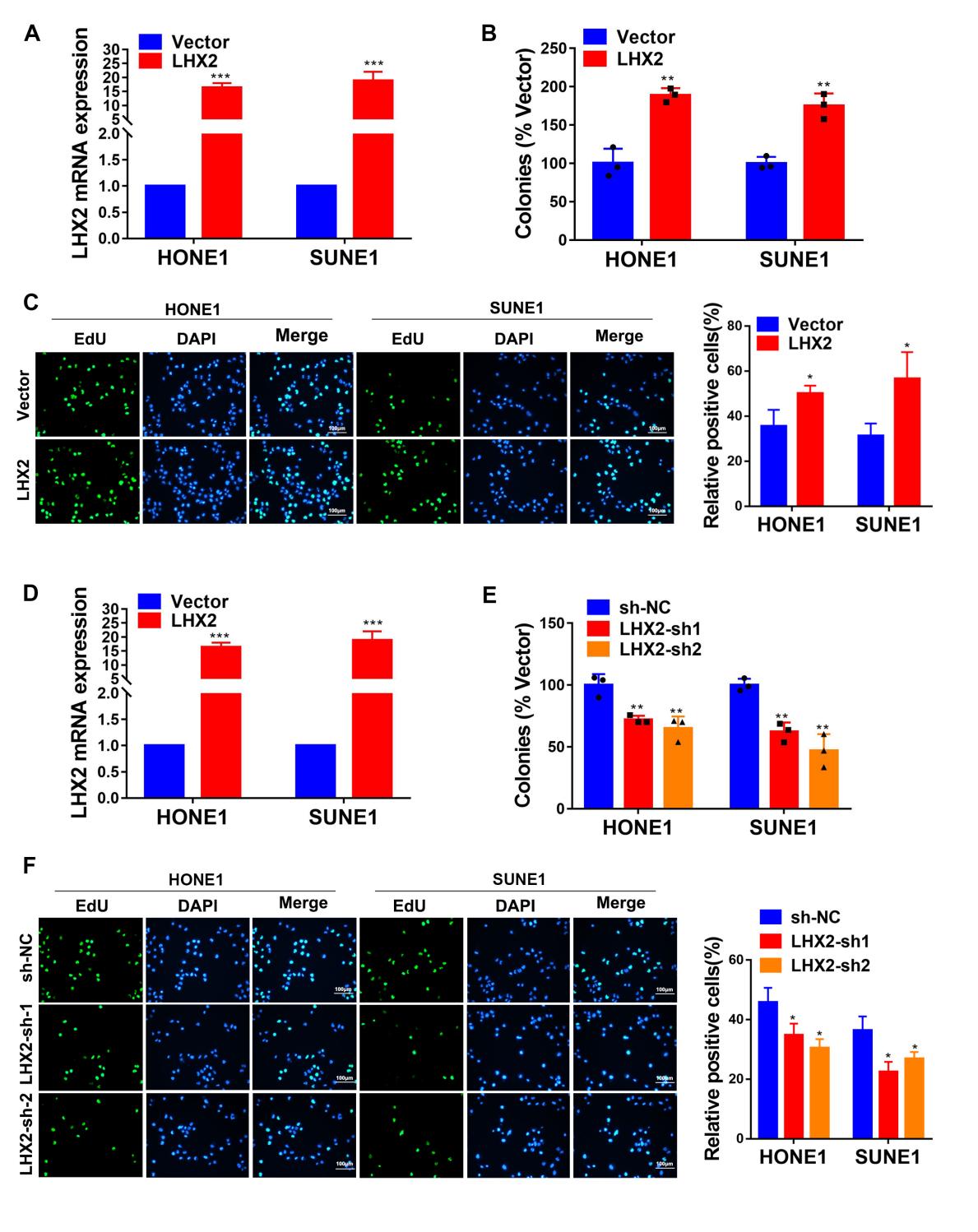
**

**Figure S3. LHX2 promotes NPC cells growth *in vitro.***

(A) RT-qPCR analysis of LHX2 mRNA expression in HONE1 and SUNE1 cells stably overexpressing LHX2. (B) The [quantitative](C:/Users/11769/AppData/Local/youdao/dict/Application/8.7.0.0/resultui/html/index.html#/javascript:;) [statistics](C:/Users/11769/AppData/Local/youdao/dict/Application/8.7.0.0/resultui/html/index.html#/javascript:;) of colonies of HONE1 and SUNE1 cells stably overexpressing LHX2. (C) The EdU staining of HONE1 and SUNE1 cells stably overexpressing LHX2. (D) RT-qPCR analysis of LHX2 mRNA expression in HONE1 and SUNE1 cells transfected with LHX2 shRNAs or control. (E) The [quantitative](C:/Users/11769/AppData/Local/youdao/dict/Application/8.7.0.0/resultui/html/index.html#/javascript:;) [statistics](C:/Users/11769/AppData/Local/youdao/dict/Application/8.7.0.0/resultui/html/index.html#/javascript:;) of colonies of HONE1 and SUNE1 cells transfected with LHX2 shRNAs or control. (F) EdU staining of HONE1 and SUNE1 cells transfected with shRNAs or control. Data shown as mean ± SD. Each experiment was independently repeated at least three times. **p* < 0.05, ***p* < 0.01, ****p* < 0.001.

**Fig. S4**

**
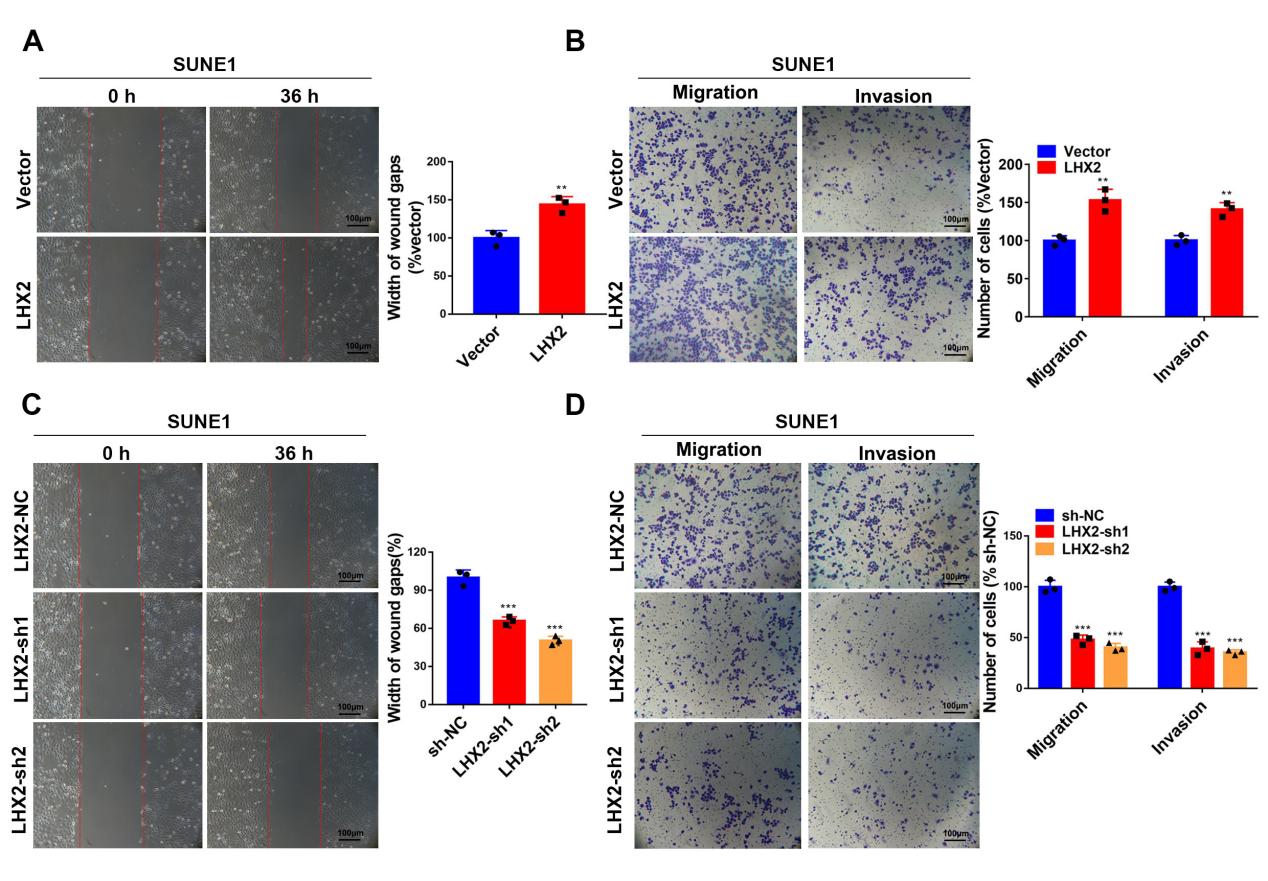
**

**Figure S4. LHX2 promotes SUNE1 cells migration and invasion.**

(A) Wound healing assays of SUNE1 cells stably overexpressing LHX2. (B) Transwell migration and invasion assays of SUNE1 cells stably overexpressing LHX2. (C) Wound healing assay of SUNE1 cells transfected with LHX2 shRNAs or control. (D) Transwell migration and invasion assays of SUNE1 cells transfected with LHX2 shRNAs or control. Each experiment was independently repeated at least three times. Data shown as mean ± SD. **p* < 0.05, ***p* < 0.01.

**Fig. S5**

**
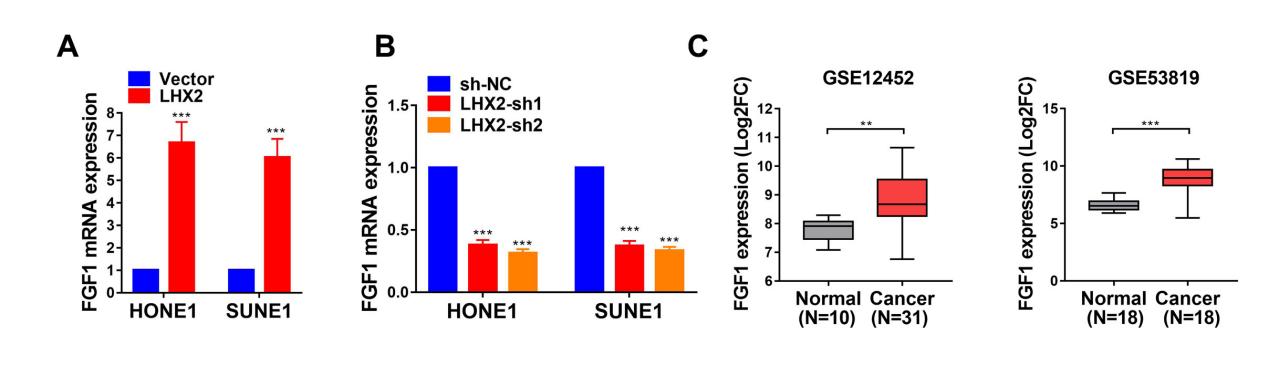
**

**Figure S5. LHX2 positively regulation FGF1 expression in NPC.**

(A) RT-qPCR analysis of FGF1 mRNA expression in HONE1 and SUNE1 cells overexpressing LHX2. Each experiment was independently repeated at least three times. (B) RT-qPCR analysis of FGF1 mRNA expression in HONE1 and SUNE1 cells transfected with LHX2 shRNAs or control. Each experiment was independently repeated at least three times. (C) FGF1 mRNA expression level is higher in nasopharyngeal carcinoma tissues than in normal tissues. The data analyzed were from GSE12452 and GSE53819 datasets. Data shown as mean ± SD. ***p* < 0.01, ****p* < 0.001.

**Fig. S6**

**
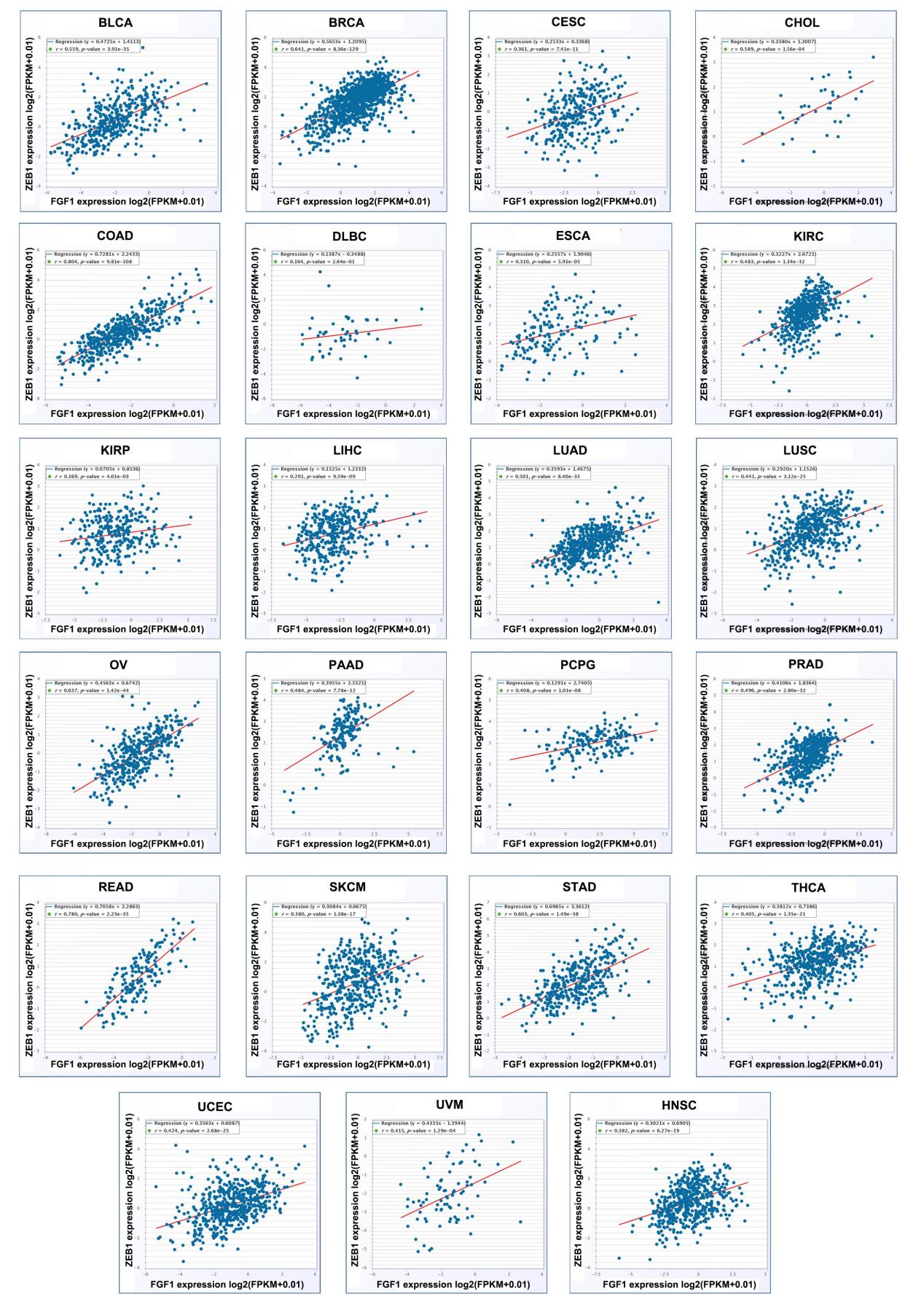
**

**Figure S6. FGF1 mRNA expression is positively correlated with ZEB1 mRNA expression in pan-cancers.**

Bladder urothelial carcinoma (BLCA); Breast invasive carcinoma (BRCA); Cervical squamous cell carcinoma and endocervical adenocarcinoma (CESC); Cholangio carcinoma (CHOL); Colon adenocarcinoma (COAD); Lymphoid Neoplasm Diffuse Large B-cell Lymphoma (DLBC); Esophageal carcinoma (ESCA); Kidney renal clear cell carcinoma (KIRC); Kidney renal papillary cell carcinoma (KIRP); Liver hepatocellular carcinoma (LIHC); Lung adenocarcinoma (LUAD); lung squamous cell carcinoma (LUSC,); Ovarian serous cystadenocarcinoma (OV); Pancreatic adenocarcinoma (PAAD)；Pheochromocytoma and Paraganglioma (PCPG); Prostate adenocarcinoma (PRAD); Rectum adenocarcinoma (READ); Skin cutaneous melanoma (SKCM); stomach adenocarcinoma (STAD); Thyroid carcinoma (THCA); Uterine corpus endometrial carcinoma (UCEC); Uveal Melanoma (UVM); Head and Neck squamous cell carcinoma (HNSC)

**Fig. S7**

**
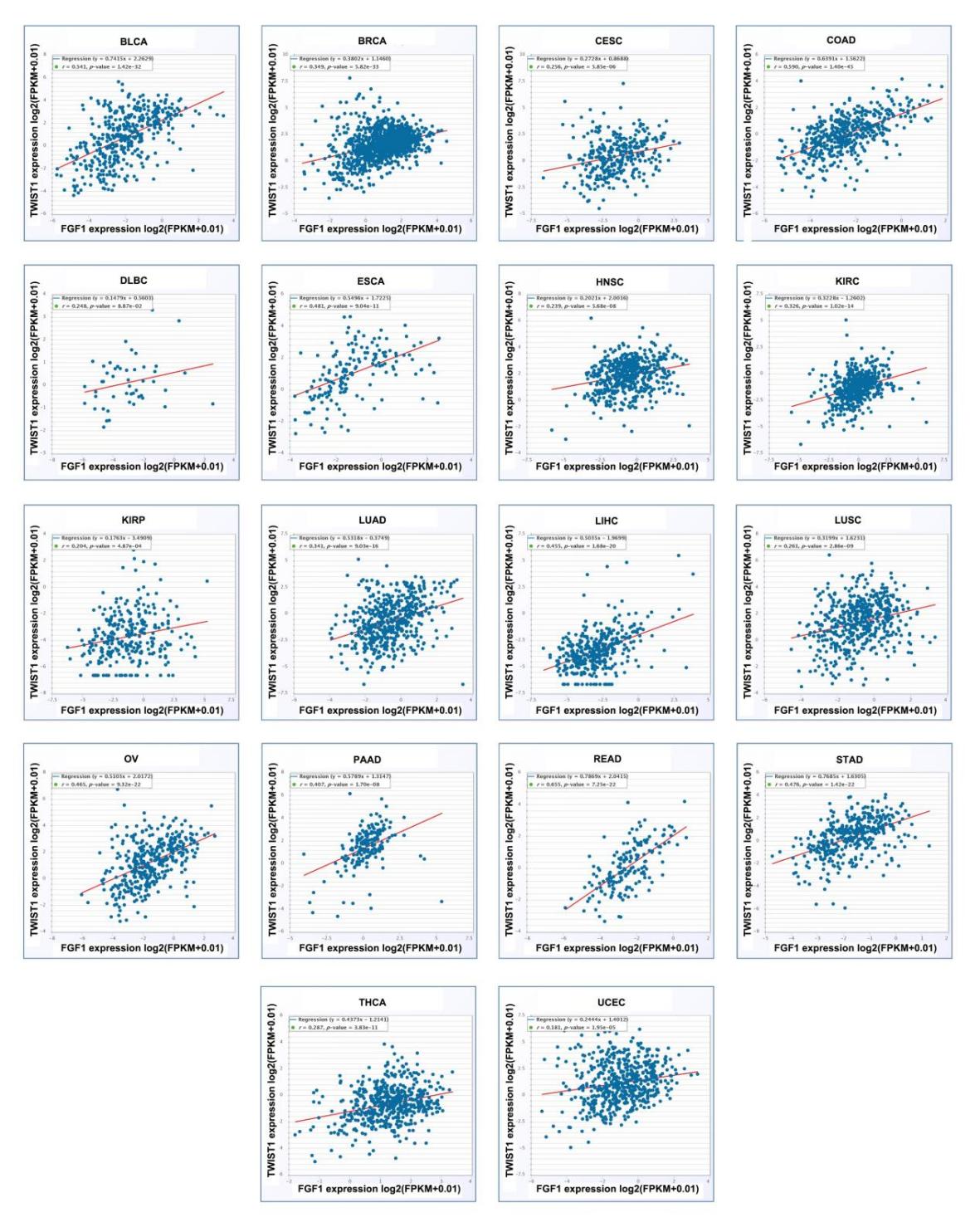
**

**Figure S7. FGF1 mRNA expression is positively correlated with TWIST1 mRNA expression in pan-cancers.**

Bladder urothelial carcinoma (BLCA); Breast invasive carcinoma (BRCA); Cervical squamous cell carcinoma and endocervical adenocarcinoma (CESC); Colon adenocarcinoma (COAD); Lymphoid Neoplasm Diffuse Large B-cell Lymphoma (DLBC); Esophageal carcinoma (ESCA); Head and Neck squamous cell carcinoma (HNSC); Kidney renal clear cell carcinoma (KIRC); Kidney renal papillary cell carcinoma (KIRP); Liver hepatocellular carcinoma (LIHC); lung adenocarcinoma (LUAD); Lung squamous cell carcinoma (LUSC); Ovarian serous cystadenocarcinoma (OV); Pancreatic adenocarcinoma (PAAD)；Rectum adenocarcinoma (READ); Skin cutaneous melanoma (SKCM); stomach adenocarcinoma (STAD); Thyroid carcinoma (THCA); Uterine corpus endometrial carcinoma (UCEC).

**Fig. S8**

**
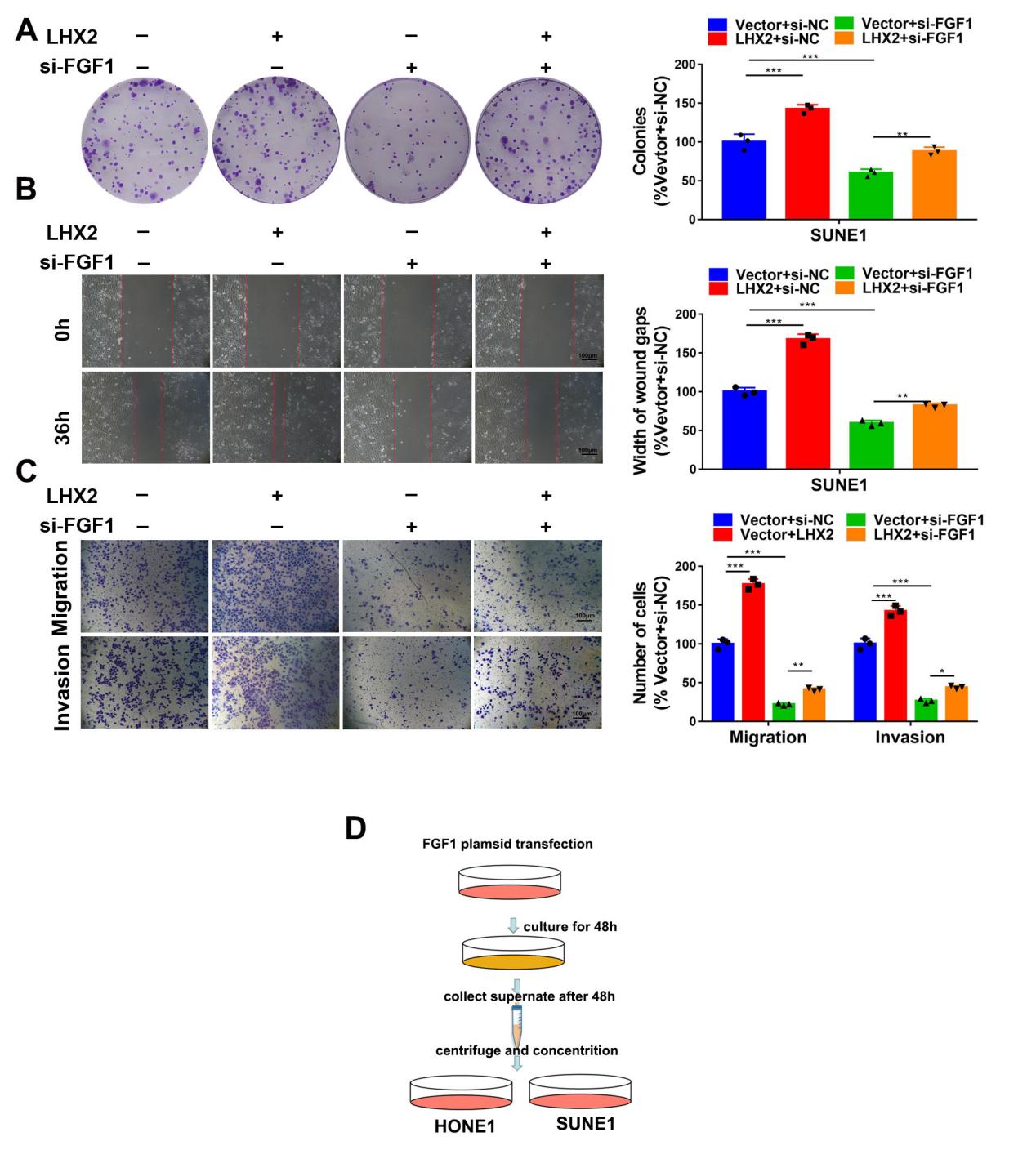
**

**Figure S8. Silencing FGF1 blocks LHX2-induced proliferation, migration and invasion in SUNE1 cells**

(A-C) FGF1 siRNA was transfected into SUNE1 cells stably overexpressing LHX2 or containing the empty vector. Colony formation assay (A), wound healing assay (B) and transwell migration and invasion assays(C). Each experiment was independently repeated at least three times. (D) A schematic diagram for the FGF1-CM preparation and cell treatment. **p* < 0.05, ***p* < 0.01, ****p* < 0.001.

**Fig. S9**

**
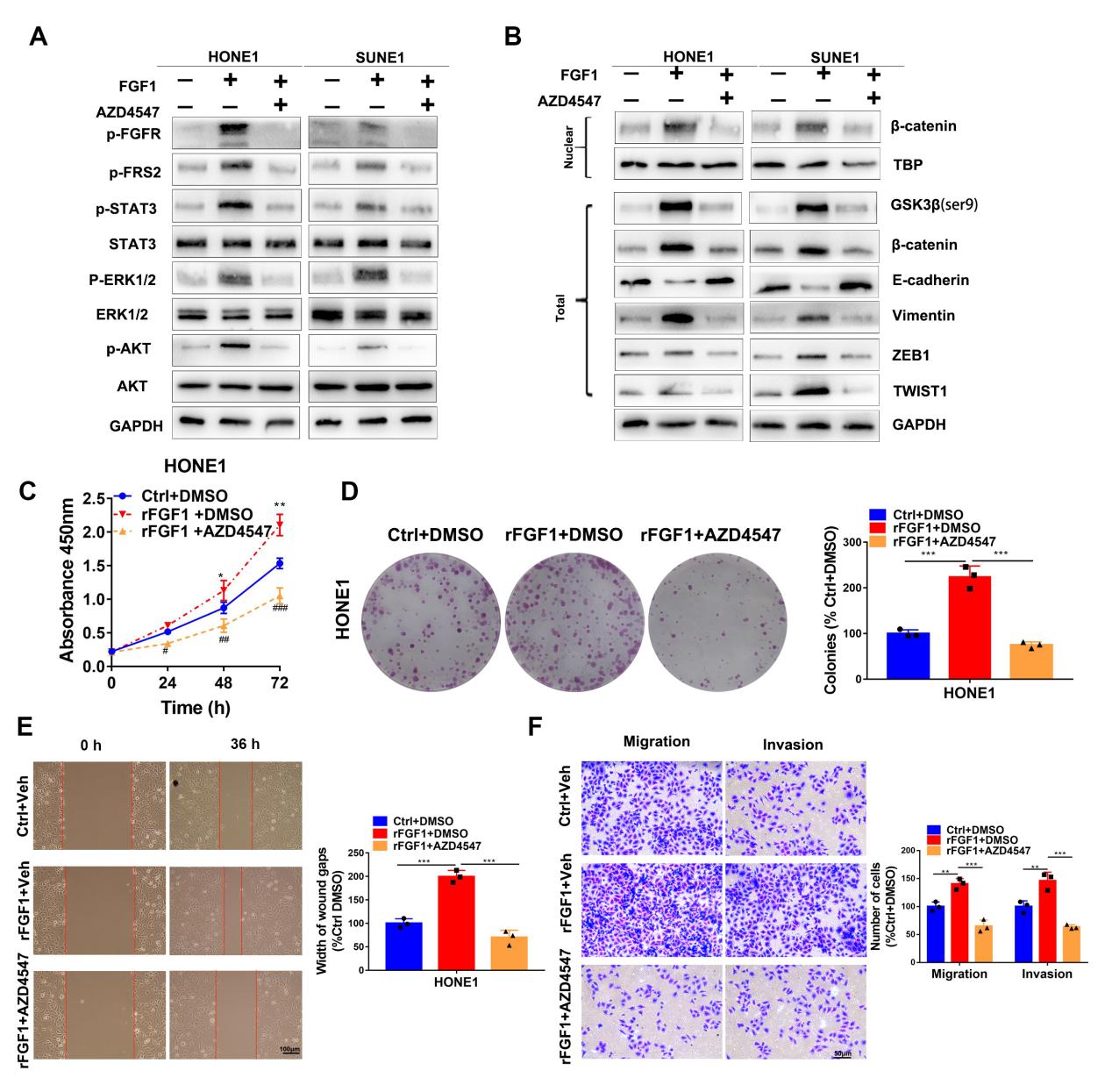
**

**Figure S9. AZD4547 blocks FGF1/FGFR signaling pathway in NPC.**

(A and B) HONE1 and SUNE1 cells were serum-starved, treated with AZD4547 (2μM）or DMSO, and stimulated with FGF1 (20 ng/ml) for 48h. Whole cell lysates were subjected to Western blot analysis.(C-F) HONE1cells were treated with AZD4547(100nM) or DMSO, and stimulated with FGF1 (20 ng/ml) for 48h. The cells were harvested and subjected to CCK8 assay(C), colony formation assay (D), wound healing assay (E) and Transwell migration and invasion assays (F). Each experiment was independently repeated at least three times. Data are shown as means ± SD. *p < 0.05, **p < 0.01, ***p < 0.001.

**Fig. S10**

**
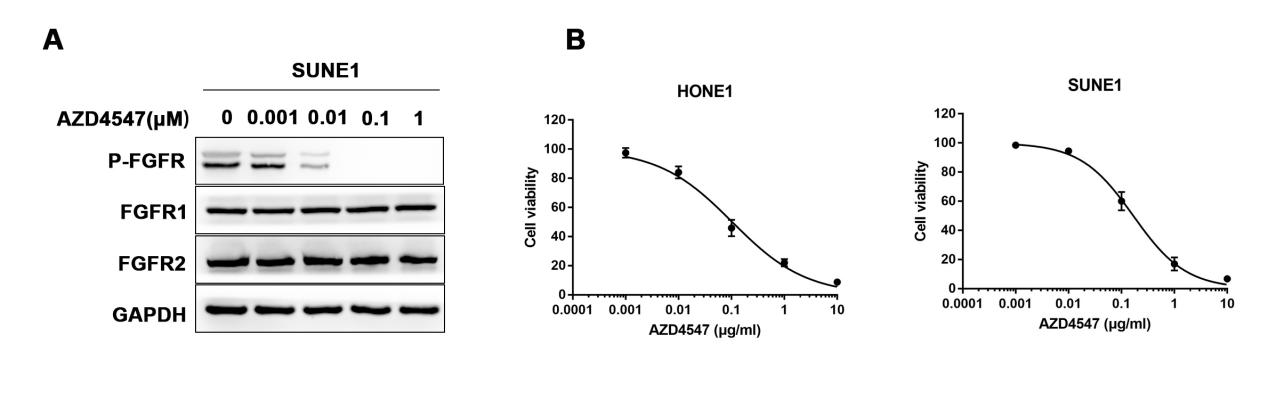
**

1. SUNE1 cells were incubated for 4 hours of AZD4547 at different concentration and then lysed and immunoblotted for the indicated proteins.(B) HONE1 and SUNE1 cells were treated with different concentration of AZD4547 for 72h, the cell viability were examined by CCK-8 assay.

**Fig.S11**

**
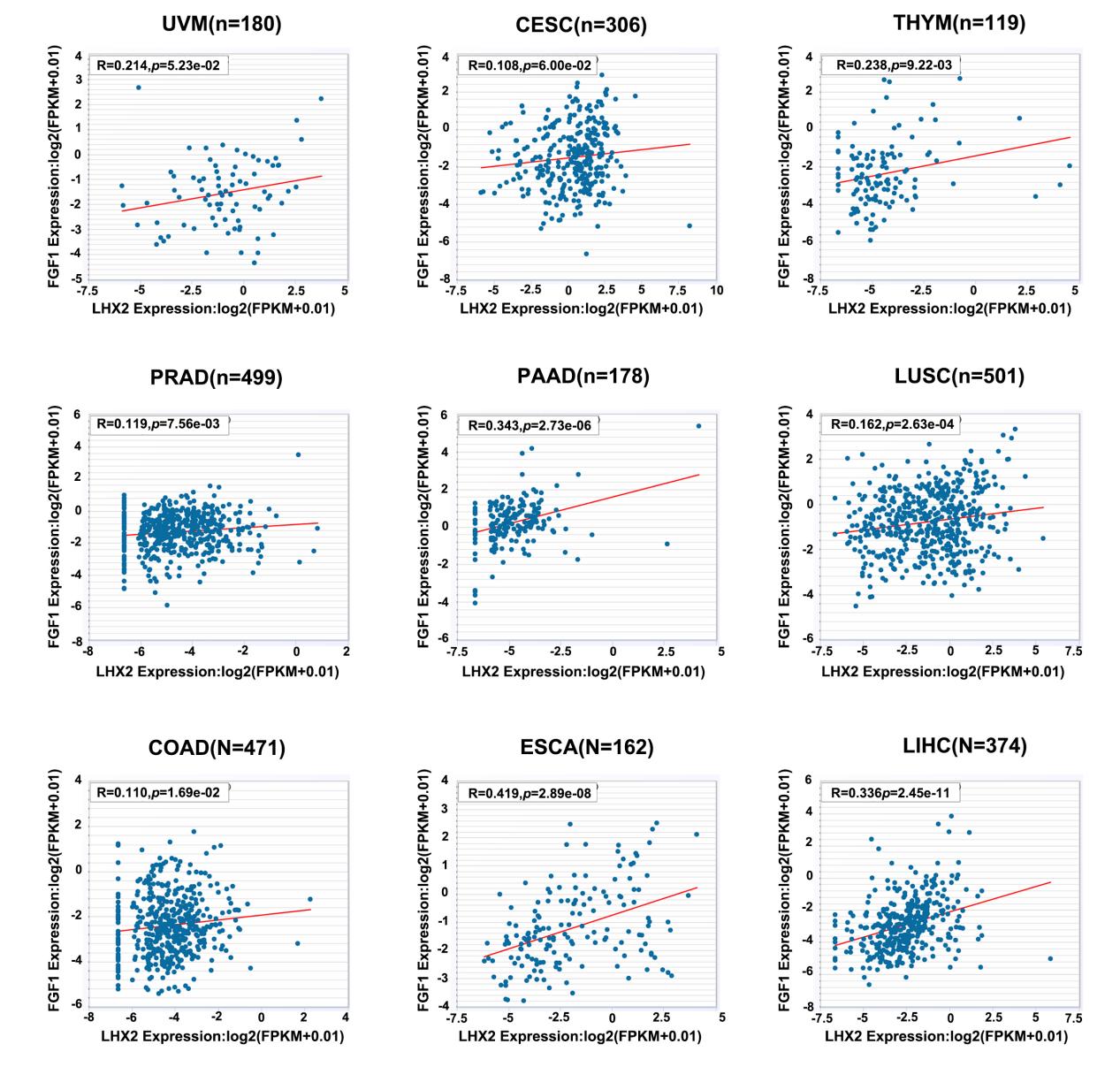
**

**Figure S11. LHX2 mRNA expression is positively correlated with FGF1 mRNA expression in pan-cancer.**

Uveal Melanoma (UVM); Cervical squamous cell carcinoma and endocervical adenocarcinoma (CESC); Thymoma (THYM); Prostate adenocarcinoma (PRAD); Pancreatic adenocarcinoma (PAAD); Lung squamous cell carcinoma (LUSC); Colon adenocarcinoma (COAD); Esophageal carcinoma (ESCA); Liver hepatocellular carcinoma (LIHC).
